# Phosvitin Derived Phospho-Peptides Show Better Osteogenic Potential than Intact Phosvitin in MC3T3-E1 Osteoblastic Cells

**DOI:** 10.3390/nu12102998

**Published:** 2020-09-30

**Authors:** Subhadeep Chakrabarti, Jiandong Ren, Jianping Wu

**Affiliations:** 1 Department of Agricultural, Food and Nutritional Science, University of Alberta, Edmonton, AB T6G2P5, Canada; subhadee@ualberta.ca (S.C.); jr2@ualberta.ca (J.R.); 2 Cardiovascular Research Centre and Women and Children’s Health Research Institute, University of Alberta, Edmonton, AB T6G2P5, Canada

**Keywords:** osteoblast, phosvitin, phosphopeptides, phosphoprotein, RANKL, ECM

## Abstract

Phosphorylated proteins from food sources have been investigated as regulators of bone formation with potential benefits in treating osteoporosis. Egg, a cheap and nutritious food, is also the source of various proteins and bioactive peptides with applications in human health. Egg yolk is rich in phosvitin, the most phosphorylated protein in nature. Phosvitin has been shown to improve bone health in experimental animals, although the molecular mechanisms and its specific effects on bone-forming osteoblastic cells are incompletely understood. Previous work in our group has identified pancreatin-generated phosvitin phospho-peptides (PPP) as a potential source for bioactive peptides. Given this background, we examined the roles of both phosvitin and PPP in the function of osteoblastic cells. Our results demonstrated their potential to improve bone health by promoting osteoblast differentiation and proliferation, suppressing osteoclast recruitment and the deposition of extracellular matrix, although PPP appeared to demonstrate superior osteogenic functions compared to phosvitin alone.

## 1. Introduction

There is growing interest in using food-derived proteins and bioactive peptides for improving human health and treating disease conditions due to their perceived safety and acceptability compared to pharmaceutical agents [1,2]. Phosphoproteins, rich in phospho-serine and phospho-threonine residues, are the subject of research due to their antioxidant, anti-inflammatory and metal-binding properties, with potential applications in health [3,4]. In fact, both native phosphoproteins as well as their enzymatic hydrolysates, containing an array of diverse phospho-peptides, are being evaluated for future usage as nutraceuticals or functional foods [5]. Given the abundance of phosphorylated matrix proteins within bone, a number of phosphoproteins have been suggested for improving bone health under physiological and pathological conditions [6]. Studies on casein-derived phospho-peptides suggest their potential beneficial roles in bone formation. These results indicate the potential therapeutic applications of phospho-peptides in the management of osteoporosis, a debilitating condition affecting millions worldwide [7,8]. In normal bone, there is a perpetual remodeling involving new bone formation by osteoblasts and bone degradation by osteoclasts [9]. This balance is shifted towards increased bone loss in osteoporosis and few pharmacological remedies (e.g., bisphosphonate) are available to successfully manage this condition [10]. However, the associated side-effects, such as nausea, abdominal pain and headache, can be severe and hinder their long-term use [11].

Egg is an affordable and widely used food item [12]. Not only is egg a great source of animal protein in the diet, its constituent proteins and their bioactive peptides have been studied for numerous biological actions, such as antihypertensive and anti-inflammatory effects, by numerous research groups, including previous work from our laboratory [13,14,15,16,17]. Egg yolk is a rich source of phosvitin, a 35–42 KDa phosphoprotein, that is considered to be the most phosphorylated protein in nature [18]. For comparison, the milk phosphoprotein casein contains less than 15 phospho-serine residues per molecule, while phosvitin could contain as many as 123 per molecule, giving it an extraordinary degree of phosphorylation, reflected in its biological properties [4,19].

Surprisingly, phosvitin is often considered nutritionally negative, as its strong metal-binding capacity may adversely affect the intestinal absorption of minerals such as iron and calcium from dietary sources [20]. This perceived harmful action might have contributed to the limited research on its applications in human health to date. However, a recent study has demonstrated the osteogenic potential of phosvitin in a mouse calvarial bone culture system [21]. Phosvitin was found to enhance bone growth (by differentiation of the pro-osteogenic osteoblasts) as well as suppress bone degradation (by inhibiting the recruitment of bone-degrading osteoclasts) under these conditions. However, the exact molecular mechanisms of phosvitin action on bone-producing osteoblastic cells remain incompletely understood. While this study offers hope for harnessing the osteogenic potential of phosvitin, the therapeutic usage of phosvitin is still limited by additional factors, such as its limited digestibility, which may hamper its absorption from the intestine and severely limit its biological activity in vivo [21]. Thus, alternatives to using native phosvitin are an attractive proposition, and include formulating preparations rich in lower molecular weight (MW) phospho-peptides derived from enzymatically hydrolyzed phosvitin.

Previous work from our research group has developed methods of phosvitin extraction from chicken egg yolks and phosvitin phospho-peptide (PPP) preparation using pancreatin, and the key PPPs were characterized [22,23]. The study aimed to evaluate the osteogenic potential of both native phosvitin and PPP on MC3T3-E1 cells, a widely used model system for osteoblasts. Our study involved determining the effects of these proteins on markers of osteoblast differentiation, the proliferation and ECM deposition by these cells, as well as their potential to prevent the recruitment of bone-degrading osteoclasts under these conditions.

## 2. Materials and Methods 

### 2.1. Reagents

Dulbecco’s phosphate buffered saline (PBS) and dithiothreitol (DTT) were bought from Sigma Chemical Co. (St. Louis, MO, USA). Dihydroethidium (DHE), DMEM and fetal FBS were obtained from Gibco/Invitrogen (Carlsbad, CA, USA). Type 1 Collagenase used for cell splitting was purchased from Worthington Biochemical Corporation (Lakewood, NJ, USA). Triton-X-100 was from VWR International (West Chester, PA, USA).

### 2.2. Cell Culture

The MC3T3-E1 cells (subclone 4, ATCC CRL-2593), a murine osteoblastic cell line, were purchased from ATCC (Manassas, VA, USA) and cultured in Dulbecco’s modified Eagle medium (DMEM) supplemented with 10% heat-inactivated fetal bovine serum (FBS) and penicillin-streptomycin in an incubator under 95% air and 5% CO_2_. All experiments were performed on 80–90% confluent cells grown in tissue culture-grade plastic 48-well plates. The cells were incubated with phosvitin, PPP or lactoferrin (the positive control for inducing osteogenic effects) for different time periods prior to western blotting, immunofluorescence or BrDU incorporation. Treatment of the cells with the highest dose of phosvitin and PPP for 72 h did not cause increases in cell death, as determined by Alamarblue staining (Supplement, Figure S1), according to the manufacturer’s instruction and previously described [24].

### 2.3. Preparation of Phosvitin and PPP 

The isolation of phosvitin from chicken egg yolks and the preparation of pancreatin-generated PPP were carried out as described in prior publications from our group [22,23].

### 2.4. Western Blotting

The cells were treated with varying concentrations of phosvitin, PPP or 0.5 mg/mL lactoferrin for 72 h. At the end of the incubation period, the culture medium was removed and the cells lysed in boiling hot Laemmle’s buffer containing 50 μM dithiothreitol (DTT, a reducing agent) and 0.2% Triton-X-100 to prepare samples for western blot, as described before [25]. These cell lysates were run in SDS-PAGE, blotted to nitrocellulose membranes and immunoblotted with antibodies against alkaline phosphatase (ALP; mouse monoclonal antibody from Santa Cruz Biotechnologies, Santa Cruz, CA, USA), RANKL (rabbit polyclonal antibody from Santa Cruz Biotechnologies) and the loading control α-tubulin (rabbit polyclonal antibody from Abcam, Cambridge, MA, USA). Anti-tubulin was used at 0.4 μg/mL, while the others were used at 1 μg/mL. Goat anti-rabbit and Donkey anti-mouse fluorochrome-conjugated secondary antibodies were purchased from Licor Biosciences (Lincoln, NB, USA). The protein bands were detected by a Licor Odyssey BioImager and quantified by densitometry using corresponding software (Licor Biosciences). Each band of ALP or RANKL was normalized to its corresponding band of loading control. Cell lysates from untreated cells were loaded onto every gel. The results were expressed as percentages of the corresponding untreated control results.

### 2.5. ELISA 

Following 72 h incubation with varying concentrations of phosvitin or PPP, cell free culture supernatants were collected by centrifugation (12,000× *g*, 10 min, 4 °C). The RANKL content of such supernatants was determined by a commercially available ELISA kit (*ab100749-RANKL* from Abcam, Cambridge, MA, USA).

### 2.6. Immunofluorescence

The immunofluorescence studies were performed similarly to our previous studies [26,27]. Briefly, cells were fixed in 4% formalin, permeabilized with 0.1% Triton-X-100 in PBS, blocked by 1% bovine serum albumin (BSA) in PBS and immunostained through overnight incubation with a rabbit polyclonal antibody against type I collagen (Novus Biologicals, Littleton, CO, USA). Cells were treated with Alexa Fluor546 (red) conjugated goat anti-rabbit secondary antibody (Molecular Probes, Eugene, OR, USA) for 30 min in the dark. Nuclei were stained with the Hoechst33342 nuclear dye (1:10,000; Molecular Probes Eugene, OR, USA) for 10 min. After washing to remove unbound antibody/dye, the immunostained cells were observed under an Olympus IX81 fluorescence microscope (Olympus, Tokyo, Japan). Images were obtained using Metamorph imaging software (Molecular Devices, Sunnyvale, CA, USA). Mean fluorescence intensity was calculated from the intensity of the red fluorescent signal (determined by Adobe Photoshop Elements 2.0 software; Adobe Systems Inc., San Jose, CA, USA) from 3 randomly selected fields per group.

### 2.7. BrDU Incorporation Assay

The cells were plated on 48-well tissue culture plates in Dulbecco’s Modified Eagle Medium (DMEM) with 10% fetal bovine serum (FBS) and incubated for 4 h. Afterwards, the medium was changed and different doses of phosvitin or PPP were added. Lactoferrin was also used as a positive control. Following 24 h incubation, the cells were washed and placed in fresh quiescing medium (DMEM with 1% FBS) containing 1% bromodeoxyuridine (BrDU; Invitrogen, Carlsbad, CA, USA) for 1 h. The cells were then fixed in 70% ethanol (20 min), treated with 1N hydrochloric acid (HCl) for antigen exposure (also 20 min), permeabilized with 0.1% Triton-X-100 in phosphate buffered saline (5 min) and blocked in 1% bovine serum albumin in phosphate buffered saline (60 min) prior to incubation with mouse monoclonal antibody against BrDU (1:1000; Cell Signaling, Beverly, MA, USA) at 4 °C. All the steps prior to the addition of primary antibody were performed at room temperature. Following overnight incubation with the primary antibody, the cells were treated with anti-mouse secondary antibody (Molecular Probes, Eugene, OR, USA) for 30 min in the dark. Nuclei were stained with the Hoechst33342 nuclear dye (Molecular Probes). Cells were visualized under an Olympus IX81 fluorescent microscope (Carson Scientific Imaging Group; Markham, ON, Canada) as described before. For each data point, 3 random fields were chosen. The percentage of nuclei positive for BrDU staining was noted in each field and the mean calculated.

### 2.8. Statistics

All data are presented as the mean ± SEM (standard error of mean) of between 4 and 7 independent experiments. The data are expressed as percentage of negative control (untreated cells). Data were analyzed using one-way analysis of variance (ANOVA) with Dunnett’s post-hoc test for comparisons to control. The PRISM 6 statistical software (GraphPad Software, San Diego, CA, USA) was used for the analyses. *p* < 0.05 was considered significant.

## 3. Results

### 3.1. Phosvitin Induces Cellular Markers of Osteoblast Differentiation

**Figure 1:**
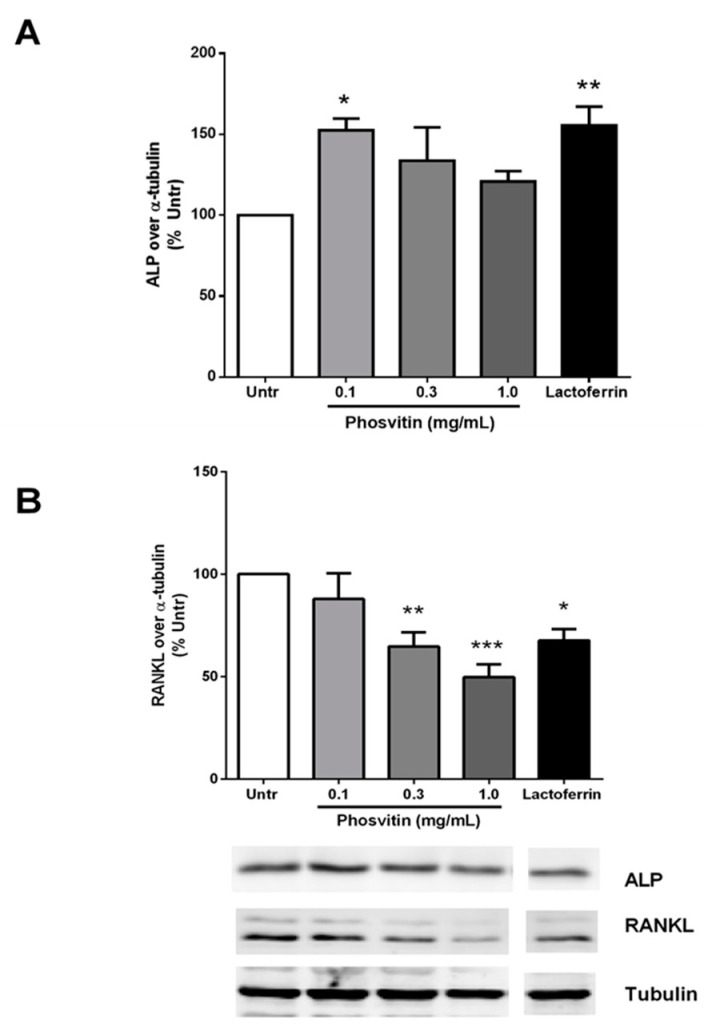
Phosvitin increases alkaline phosphatase (ALP) and decreases receptor activator of nuclear factor kappaB ligand (RANKL) in osteoblastic cells. Confluent monolayers of MC3T3-E1 cells were treated for 72 h with different concentrations (0.1–1.0 mg/mL) of phosvitin or lactoferrin (0.5 mg/mL) prior to being lysed and immunoblotted for ALP (**A**) and RANKL (**B**). A representative set of immunoblots is shown. Data are mean ± SEM from 5–6 independent experiments. *, ** and *** indicate *p* < 0.05, *p* < 0.01 and *p* < 0.001 respectively, as compared to the untreated control group.

To determine the effects of phosvitin on the differentiation of osteoblastic cells, MC3T3-E1 cells were incubated in the presence of varying concentrations of phosvitin for 72 h. The western blotting of cell lysates demonstrated that low-dose (0.1 mg/mL) phosvitin enhanced the expression of ALP significantly, while higher doses had no effect (Figure 1A). The elevation of ALP is usually associated with the activation of osteogenic activities [28]. In contrast, only the higher (0.3 and 1.0 mg/mL) but not the lower (0.1 mg/mL) doses of phosvitin significantly reduced the cellular levels of the osteoclast recruiter protein called receptor activator of nuclear factor kappaB ligand (RANKL; Figure 1B). The effects of phosvitin were comparable to those induced by lactoferrin (0.5 mg/mL), the positive control for osteogenic differentiation. As RANKL acts as a chemoattractant towards osteoclasts and helps in their differentiation (and hence bone degradation), secreted levels of RANKL are critical in promoting bone turnover [29,30]. These results establish the role of phosvitin as an inducer of differentiation in osteoblastic cells.

### 3.2. PPP also Induces Cellular Markers of Osteoblast Differentiation

**Figure 2:**
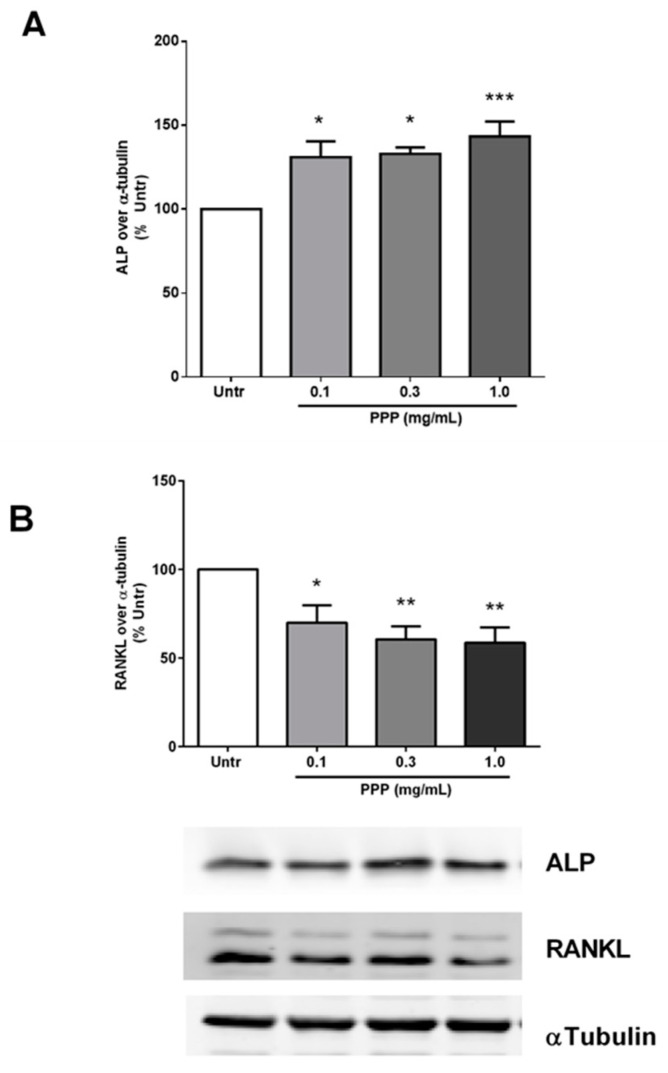
Phosvitin phospho-peptide (PPP) increases ALP and decreases RANKL in osteoblastic cells. Confluent monolayers of MC3T3-E1 cells were treated for 72 h with different concentrations (0.1–1.0 mg/mL) of PPP prior to being lysed and immunoblotted for ALP (**A**) and RANKL (**B**). A representative set of immunoblots is shown. Data are mean ± SEM from 5–6 independent experiments. *, ** and *** indicate *p* < 0.05, *p* < 0.01 and *p* < 0.001 respectively, as compared to the untreated control group.

Given the limited bioavailability of the intact phosvitin protein at its putative sites of action, we next used the pancreatin-generated hydrolysate containing phosvitin phospho-peptides (PPP) for treating MC3T3-E1 cells. Incubation with PPP for 72 h caused significant increases in ALP (Figure 2A) and corresponding decreases in RANKL (Figure 2B) in these cells. Both effects were exerted at all the concentrations tested (0.1–1.0 mg/mL) in contrast to the results with phosvitin. Given these findings, we decided to use only two doses (0.1 And 1.0 mg/mL) in all our subsequent studies.

### 3.3. Both Phosvitin and PPP Attenuate RANKL Release from Osteoblasts

**Figure 3:**
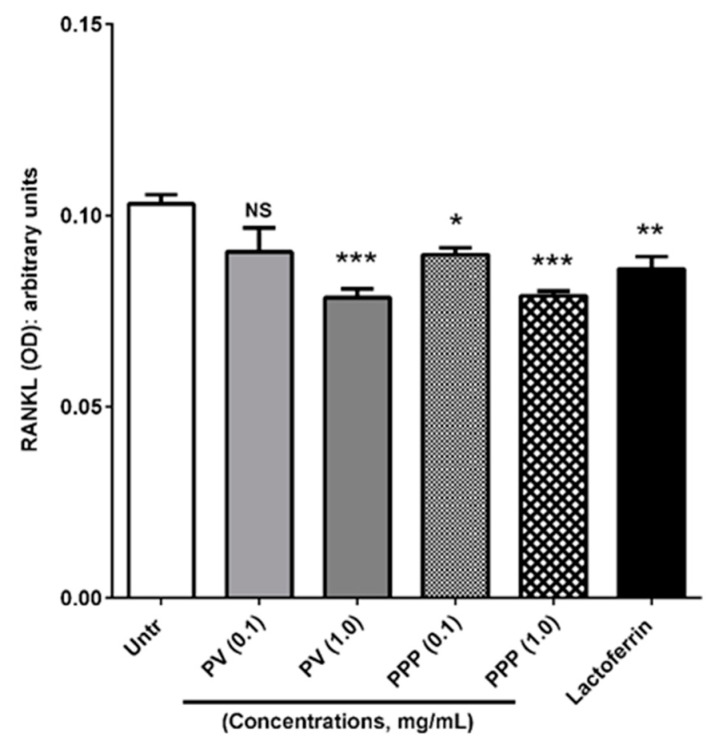
Both phosvitin and PPP reduce the levels of secreted RANKL from osteoblastic cells. Confluent monolayers of MC3T3-E1 cells were treated for 72 h with different concentrations (0.1 and 1.0 mg/mL) of phosvitin (PV) or PPP prior to collection of cell-free culture supernatants. These supernatants were analyzed for RANKL content by a commercially available ELISA kit. Lactoferrin (0.5 mg/mL) was used as a positive control. Data expressed as mean ± SEM of optical density (OD) values from 4 independent experiments. NS, *, ** and *** indicate ‘not significant’, *p* < 0.05, *p* < 0.01 and *p* < 0.001 respectively, as compared to the untreated control group.

To determine the effects of phosvitin and PPP on RANKL release from MC3T3-E1 cells, we collected cell-free culture supernatants following 72 h of incubation. An immunoassay of these supernatants showed a significant decrease in secreted RANKL by the higher dose (but not lower dose) of phosvitin, while both concentrations of PPP caused a similar decrease (Figure 3). Lactoferrin, used as a positive control in these studies, also reduced RANKL release as expected [31]. These results showed the potential for phosvitin and PPP as regulators of bone turnover similar to lactoferrin.

### 3.4. PPP, But Not Phosvitin Alone, Increases Type I Collagen in Osteoblasts

**Figure 4:**
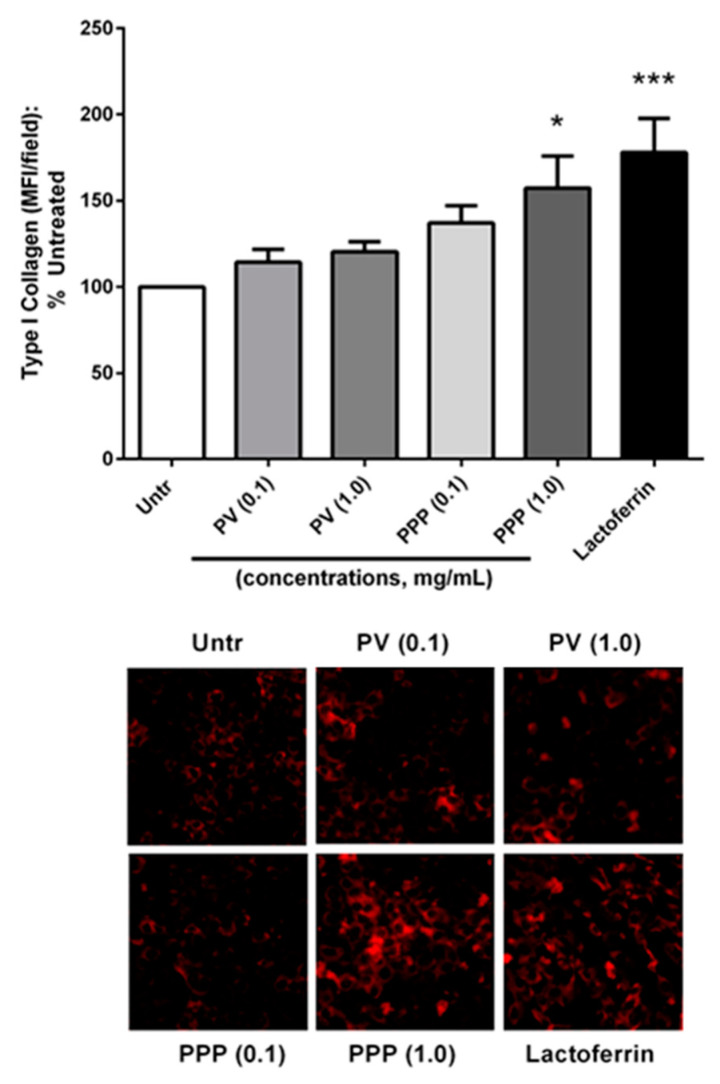
PPP but not phosvitin increase type I collagen levels in osteoblastic cells. Confluent monolayers of MC3T3-E1 cells were treated for 72 h with different concentrations (0.1 and 1.0 mg/mL) of phosvitin (PV) or PPP prior to being fixed, permeabilized and immunostained for type I collagen. Mean fluorescence intensity was measured as the average of total fluorescent intensity from 3 randomly selected fields per group and expressed as a percentage of the untreated control. Data are mean ± SEM from 5–6 independent experiments. * and *** indicate *p* < 0.05 and *p* < 0.001 respectively, as compared to the untreated control group.

As they differentiate, osteoblasts also contribute to the deposition of extracellular matrix (ECM) proteins, such as type I collagen, which form the structural components of bone. We evaluated the effects of phosvitin and PPP on these processes by immunostaining MC3T3-E1 cells for type I collagen. We found that only PPP, but not phosvitin, could significantly increase cellular levels of type I collagen (Figure 4), indicating the improved osteogenic profile of PPP compared to phosvitin alone.

### 3.5. Both Phosvitin and PPP Promote Osteoblast Proliferation

**Figure 5:**
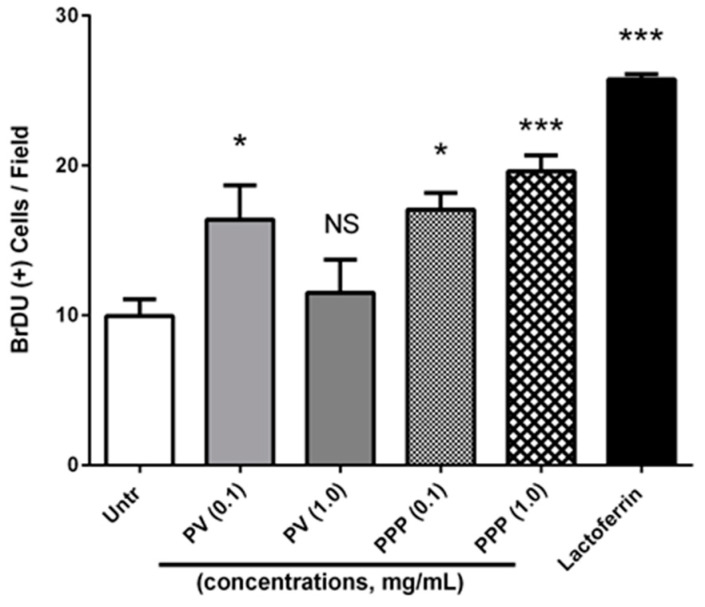
Phosvitin and PPP promote the proliferation of osteoblastic cells. Confluent monolayers of MC3T3-E1 cells were treated for 24 h with different concentrations (0.1 and 1.0 mg/mL) of phosvitin (PV) or PPP prior to being treated with the BrDU reagent, fixed, permeabilized and immunostained for BrDU. Cell nuclei were counter-stained with Hoechst33342 dye. The percentages of BrDU(+) nuclei were counted in 3 random fields per group, and their mean value determined. Data are mean ± SEM from 4–6 independent experiments. NS, *, and *** indicate ‘not significant’, *p* < 0.05, and *p* < 0.001 respectively, as compared to the untreated control group.

Finally, we studied the roles of phosvitin and PPP in stimulating the proliferation of osteoblastic cells. The determination of proliferation by BrDU incorporation assay showed that only the low-dose (0.1 mg/mL) and not the high-dose (1.0 mg/mL) phosvitin enhanced MC3T3-E1 cell proliferation, while both concentrations of PPP could stimulate proliferation (Figure 5). These results demonstrate the potential of both phosvitin and PPP as inducers of bone formation, and further indicate the improved osteogenic nature of PPP over phosvitin under these conditions.

## 4. Discussion

Our goal in this study was to study the osteogenic changes, such as alteration, in ALP and RANKL levels, and the initiation of ECM deposition. The key findings of this study were that both phosvitin and PPP could exert osteogenic and potentially anti-osteoporotic effects on osteoblastic cells in culture, although PPP appeared to have greater beneficial actions. 

Bone forms the structural framework of the body. Bone consists of two major types of cells, osteoblasts (the bone-forming cells which mature into osteocytes) and osteoclasts (the bone-degrading cells of macrophage lineage), in addition to ECM and vasculature. The hematopoietic tissue of the bone marrow is generally not considered a structural component of bone. Like many tissues, bone is in a continuous state of remodeling, involving new bone formation by osteoblasts and bone tissue breakdown by osteoclasts, which are typically held in a dynamic equilibrium under physiological states [32]. This fine balance is lost in osteoporosis when the degradation outflanks the regenerative capacity [9]. As such, therapeutic approaches promoting osteoblasts and inhibiting osteoclasts could be a viable strategy to combat this disease. Traditional therapies of osteoporosis have included supplementation with Vitamin D and calcium as well as pharmaceutical drugs like bisphosphonates [10,33,34]. Supplementation with vitamin D and calcium has been widely used for the prevention of osteoporosis as vitamin D can stimulate the synthesis of calbindin, which actively transports calcium in the intestines [35]. However, there are controversies regarding the efficacy of this intervention in cases of osteoporosis [36]. On the other hand, established anti-osteoporosis drugs such as bisphosphonates can cause adverse effects during therapy, potentially limiting their long-term usage [11]. As the treatment needed is usually long-term and the efficacy can be variable, there is an interest in developing novel therapies with fewer adverse effects for the prevention and management of osteoporosis. Food-derived proteins are typically considered to be a safer option due to their current use in diets, and as such, these are not expected to be associated with significant adverse effects.

Given the abundance of phosphorylated proteins in bone ECM and their ability to bind calcium, there has been much interest in using phosphoproteins and/or their phospho-peptide derivatives for improving bone health [37]. Apart from their ability to enhance intestinal calcium absorption, these proteins and peptides may exert additional beneficial roles through their antioxidant and/or anti-inflammatory properties. In fact, previous studies have shown the potential benefits of using phospho-peptides from casein to benefit bone health and calcium absorption [38,39,40]. While native phosphoproteins may be less desirable as therapies due to their incomplete digestion, limited absorption and potential side-effects, phospho-peptide preparations containing a number of different peptides may be able to overcome these issues [17].

While casein has been the focus of initial research involving phosphoproteins and bone health, other food-derived phosphoproteins may also demonstrate the potential to improve bone functions [6]. The egg protein phosvitin is an attractive candidate due to its unusually high degree of phosphorylation, as well as its abundant availability in a common and affordable food source [4]. However, there is still resistance to the usage of native phosvitin due to its perceived lack of digestibility and its potential to adversely affect calcium absorption in the gut [20]. However, phosvitin itself has direct osteoprotective effects, as shown in an ex vivo system of calvarial bone culture [21]. Phosvitin likely mimics the role of ascorbic acid in osteoblast cell culture to promote bone mineralization [41]. These effects may be associated with its degree of phosphorylation, which can play a critical role in the proliferation and differentiation of osteoblast cells [42]. Since enzymatic hydrolysis to generate phospho-peptides would likely avoid the perceived pitfalls of native phosvitin, PPPs are a possible approach to enhancing osteogenic and/or osteoprotective functions. Previous studies have also suggested that PPPs show enhanced bioactivities (anti-inflammation, antioxidant and calcium absorption promoting) compared to native phosvitin [43,44], further supporting the role(s) of phosphorylation in bioactive properties. In contrast, bioactive peptides lacking of phosphorylation, such as LRW derived from pea protein [45], VLPVPQK, EDVPSER, NAVPITPTL, HPHPHLSF derived from buffalo casein [46], YVEEL and YLLF derived from whey proteins [47], and IRW from egg proteins [48], were also reported to promote the proliferation of MC3T3-E1 cells, but they may not be as resistant to digestion as phospho-peptides, which therefore limits their potential applications [42]. Despite these potential benefits, the osteogenic capacity of PPP preparations is yet to be determined.

Our findings now demonstrate the capacity of both phosvitin and PPP (generated by pancreatin treatment of phosvitin) to enhance bone health in an osteoblastic cell culture system. The beneficial effects of both phosvitin and PPP were observed on markers of osteoblast function, which was accompanied by a reduction in the secreted levels of the osteoclast recruiter RANKL, as well as evidence of osteoblast proliferation. PPP, but not native phosvitin, also increased levels of type I collagen, a key component of bone ECM [49]. Taken together, these results suggested the potential of phosvitin and PPP as potential promoters of bone health.

Interestingly, PPP appeared to be superior to phosvitin in mediating many of these beneficial functions, and its effects appeared to be more consistent across different concentrations. While the molecular mechanisms underlying these effects are yet to be determined, the greater diversity of functional phospho-peptides and their probable additive (or even supra-additive) functions may explain the observed increases in osteogenic actions. Given the previously discussed shortcomings of native phosvitin and the greater likelihood of enhanced intestinal uptake of shorter peptides, PPP appears to be a viable target for future studies into bone health and the management of osteoporosis. Possible directions of future research should include studying the effects of PPP on osteoclasts and the use of already established in vivo and ex vivo models of bone growth and degradation to further validate the key findings of the current study.

## Supplementary Materials

The following are available online at https://www.mdpi.com/2072-6643/12/10/2998/s1, Figure S1, Cell viability of PV/PPP treated MC3T3-E1 cells.
