# Supplementary material for: Phosvitin Derived Phospho-Peptides Show Better Osteogenic Potential than Intact Phosvitin in MC3T3-E1 Osteoblastic Cells
Source: Nutrients. 2020 Sep 30;12(10):2998. doi: 10.3390/nu12102998 (PMC7601474; doi:10.3390/nu12102998)
Supplement: Supplementary file 1 [file nutrients-12-02998-s001.pdf]

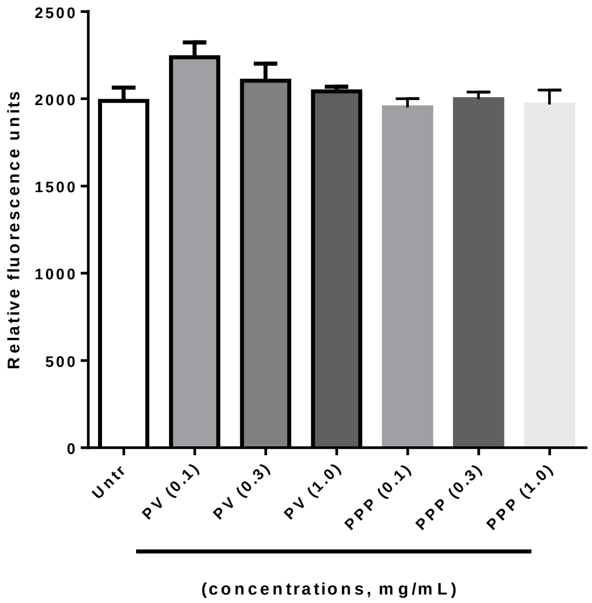

Figure 1. Cell viability of PV/PPP treated MC3T3-E1 cells. Cells were incubated in a 96 well tissue culture plate for 72 hours with different concentrations (0.1, 0.3 & 1.0 mg/mL) of phosvitin (PV) or PPP, then 20 μL Alamar blue was added for another 4 hr prior to collection of cell-free culture supernatants. The fluorescence of the medium was read with excitation wavelength of 560 nm and emission wavelength of 590 nm. Data are mean±SEM from 4 independent experiments. *, ** and *** indicate $p<0.05$, $p<0.01$ and $p<0.001$ respectively, as compared to the untreated control group.
